# Parent and child physical activity and sedentary time: Do active parents foster active children?

**DOI:** 10.1186/1471-2458-10-194

**Published:** 2010-04-15

**Authors:** Russell Jago, Kenneth R Fox, Angie S Page, Rowan Brockman, Janice L Thompson

**Affiliations:** 1 Department of Exercise, Nutrition & Health Sciences, Centre for Sport, Exercise & Health, University of Bristol, Tyndall Avenue, Bristol, BS8 1TP, UK

## Abstract

**Background:**

Physical activity has many positive effects on children's health while TV viewing has been associated with adverse health outcomes. Many children do not meet physical activity recommendations and exceed TV viewing guidelines. Parents are likely to be an important influence on their children's behaviour. There is an absence of information about the associations between parents' and children's physical activity and TV viewing.

**Methods:**

Year 6 children and their parent were recruited from 40 primary schools. Results are presented for the 340 parent-child dyads with accelerometer data that met a ≥ 3 day inclusion criteria and the 431 parent-child dyads with complete self-reported TV viewing. Over 80% of the dyads with valid TV viewing data included mothers and their child. Mean minutes of moderate to vigorous physical activity (MVPA), minutes of sedentary time per day and counts per minute were assessed by accelerometer. Self-reported hours of TV viewing were coded into 3 groups (< 2 hours per day, 2-4 hours per day and >4 hours per day. Linear and multi-nominal regression models were run by child gender to examine parent-child associations.

**Results:**

In linear regression models there was an association for the overall sedentary time of girls and their parents (t = 2.04. p = .020) but there was no association between girls' and parents' physical activity. There were no associations between parents' and boys' sedentary or physical activity time. For girls, the risk of watching more than 4 hours of TV per day, (reference = 2 hours of TV per day), was 3.67 times higher if the girl's parent watched 2-4 hours of TV per day (p = 0.037). For boys, the risk of watching more than 4 hours of TV per day, was 10.47 times higher if the boy's parent watched more than 4 hours of TV per day (p = 0.038).

**Conclusions:**

There are associations in the sedentary time of parents and daughters. Higher parental TV viewing was associated with an increased risk of high levels of TV viewing for both boys and girls. There were no associations between the time that parents and children spend engaged in physical activity.

## Background

Regular physical activity helps to prevent the development of heart disease [1], type 2 diabetes [2], obesity [3,4] and some cancers [5] and is also associated with improved mental well-being [6]. Among children and adolescents, physical activity has been associated with a lower body mass index [7] and lower mean values for cardiovascular risk factors [8-10]. Sedentary time, i.e. when people are not active has also been associated with increased adiposity among children [11]. Sedentary time does not, however, provide information about the activities in which a person is engaged and therefore there have been recent calls to focus on sedentary activities as specific behaviours that are related to health outcomes [12]. The most studied sedentary behaviour is TV viewing and higher levels of TV viewing have been associated with higher body mass among youth [7,13,14]. Many children and adolescents do not meet physical activity guidelines and exceed TV viewing recommendations [15-18]. Children's physical activity levels decline steeply with age, particularly into adolescence [19-21] with the end of primary school (10-11 years of age) being a pivotal period of change [20,22]. Therefore, in order to prevent the development of cardiovascular and associated diseases there is a need to understand the factors that influence children's physical activity and TV viewing at this key transition age.

Parents are likely to be an important influence on their children's physical activity and TV viewing behaviours [23-26]. In the UK there are now several types of family structure with many children not living with both of their biological parents. Therefore, for this paper, parent refers to the child's primary carer(s) which would usually be the child's biological parent but could also be a foster parent, grandparent or any other legal guardian. There may be several mechanisms underpinning parental impact such as parents and children sharing activities and thus engaging in activities together, parents setting examples and standards through role modelling, and providing home environments that either facilitate or prevent behaviours such as active play or TV viewing [23]. Understanding whether and/or how parent(s) influence children's physical activity and TV viewing could be important for identifying effective strategies for increasing children's physical activity.

A recent review of the associations between parent and child physical activity found that the current evidence is equivocal [23], with the majority of studies using self/parental reports of physical activity, often in small samples [23]. It is not clear if parent-child physical activity associations are evident when more robust measures are utilised or when investigated in a UK context. Therefore, the aim of this study was to examine, using objective and reliable methods, associations between the physical activity, sedentary and TV viewing patterns of 10-11 year old children and their parents.

## Methods

### Sampling and participants

This study was part of a larger project, the Bristol 3Ps Project http://www.bristol.ac.uk/enhs/research/projects/bristol3ps.html which examines the influences of peers and parents on physical activity participation in 10-11 year old children. Sampling was performed based on primary school location and the Index of Multiple Deprivation (IMD) score for school postcode. The IMD is an area level measure of deprivation produced by the UK government that includes income, health, educational and employment status [27] relative to a small geographical area around the participant's home address. IMD score therefore provides a global indicator of socioeconomic status (SES) that takes account of all the factors that are associated with SES. Higher IMD scores indicate higher levels of area deprivation i.e. lower SES. The IMDs of all state-funded schools within a 15-mile radius of the University of Bristol were obtained and divided into thirds to provide tertiles of school IMD. To provide a representative sample of local children we aimed to achieve an equal number of schools from each tertile. Fifty schools were approached and forty schools agreed to participate. The final sample approximately reflected IMD tertiles, with twelve schools from the high SES tertile group, 16 from the middle SES tertile and twelve from the low SES tertile. In total, 1684 Year 6 children and their parents were invited to take part in the study and 986 children and 539 parents provided data.

A briefing event was held at each school and all Year 6 pupils were invited to participate in a study examining parents and children's physical activity patterns. Information packs were sent home with all pupils and only those children who provided written informed parental consent took part. The self-identifying primary care provider, who will hereafter be referred to as the parent, was also invited to participate in the study and informed consent obtained. Children could participate without a parent taking part in the study. This study was approved by a University of Bristol ethics committee.

### Procedures

Parental and child physical activity and sedentary time was assessed using Actigraph accelerometers (Actigraph, Florida) which have been shown to provide accurate and reliable indices of physical activity among both children and adults [28-30]. All children wore GT1M monitors which were set to record data every 10 seconds. This short epoch was used in order to capture the intermittent nature of children's physical activity [31]. Parents wore 7164 monitors set to record at 1 minute epochs. All participants were provided with instructions on how to wear the monitor and data were collected for five complete days, including two weekend days.

Although accelerometers provide information about sedentary time, they cannot provide contextual information about what the person is doing whilst sedentary. Therefore, to provide behavioural information, the accelerometer data was supported by parent and child self-reported measures of TV viewing. TV viewing was assessed using a single question which asked both parents and children to report the number of hours per day spent watching TV (none, <1, 1-2, 2-3, 3-4, 4-5 or >5). The assessment of TV viewing via a single question has been shown to correlate (r = 0.60) with 10 days of TV diaries among young children [32]. Moreover, a recent review reported that the single item approach has the highest validity of current methodologies [33].

Child height was measured using a SECA Leicester stadiometer (HAB International, Northampton) and weight using a SECA 899 digital scale (HAB International, Northampton). Child body mass index (BMI = kg/m^2^) was calculated and converted to an age and gender specific standard deviation score (BMI SDS) [34]. Parental height and weight were self-reported and parental BMI was calculated. Household postcode was obtained via parental report and the home associated IMD obtained for all participants. Since physical activity patterns have been shown to differ by the hours of daylight available [35], the hours of daylight on the first day of data collection were also calculated for each participant using standard UK tables [36] and treated as a confounder in all analyses.

### Data reduction

Participants were included in the accelerometer analysis only if both the parent and child provided at least 3 days of valid accelerometer data. Based on the criteria used to analyse the accelerometer data in the US National Health and Nutrition Examination Survey (NHANES), periods in which 60 or more minutes of zero counts were obtained were interpreted as time when the monitor was not worn, and these periods were removed from the analysis [37]. Each day of accelerometer data was considered valid if data were obtained for at least 500 minutes. Mean counts per minute, which provides an indication of the overall volume of physical activity in which a person engages, was calculated for all participants. Mean minutes of moderate to vigorous intensity physical activity (MVPA), the intensity of activity that is recommended for optimal health [38] was also derived for both parents and children. For parents, physical activity that resulted in ≥2020 CPM was treated as minutes of MVPA [39]. For children, a criteria of 3200 CPM was used, but as the GT1M monitors yield values that are 9% different than the 7164 monitors [40], a correction factor of 0.91 was used to produce a cut-point of 2912 counts per minute or 485 counts per 10 second epoch. The accelerometer data were also used to derive minutes of sedentary time per day with thresholds of <100 CPM used for parents [41] and < 727 (799*0.91) [29] for children. We recognise that the use of accelerometer cut-points is open to debate and that the selection of childhood thresholds in particular is hotly contested [42]. We opted for the 3200 and 799 childhood cut-points because these were obtained from laboratory calorimetery among U.S. children aged 6-16 years, the most robust validation method that has been employed [29]. Moreover, by also reporting mean counts per minute we provide an indicator of physical activity that is not affected by this issue.

The American Academy of Pediatrics (AAP) have recommended that children should spend less than 2 hours per day watching television [43]. As a result researchers have often used a criteria of <2 hours of TV viewing per day to describe children meeting the AAP guideline [44] or >4 hours to indicate children who greatly exceed this public health guideline [45,46]. The coding of the both the child and parent TV viewing data in this paper was modelled on these criteria with both child and parent TV viewing placed into 3 groups (< 2 hours per day, 2-4 hours per day, and >4 hours per day). These three categories thereby provided an indication of the extent to which both the children and parents met (<2 hours per day), exceeded (2-4 hours) or greatly exceeded (>4 hours per day) the Academy of Pediatrics guidelines on television viewing for children [43].

### Analysis

Descriptive statistics including means, percents and standard deviations were calculated for all variables. Scatter-plots and Pearson correlations were used to examine the associations between parent and child accelerometer variables. As the graphs indicated a weak, linear association, linear regression models were then used to further investigate the associations between parent and child physical activity and TV viewing. As children's physical activity has been shown to differ by child gender [15,47] all analyses were performed separately for boys and girls.

Models were built to examine if there was an association between parental BMI and the key exposure variables (parental behaviour). This was done by adding an interaction term between parental BMI and the parental exposure of interest into linear regression models that also included main effects for parental BMI and the exposure variable. Initial analyses indicated that none of these interaction terms were significant, therefore all models were run for all parent-child dyads with valid data, with parental BMI treated as a potential confounder. Linear regression models were then conducted to examine the extent to which parental physical activity (counts per minute) predicted child physical activity (counts per minute). The model was adjusted for child BMI SDS, parental BMI, household IMD score and hours of daylight on the first day of data collection. As the children were recruited from schools the model was also adjusted for the clustering of participants within schools, and robust standard errors were used. This process was then repeated separately for sedentary and MVPA minutes.

Multi-nominal logistic regression models with relative risk ratios (RR) were used to examine if either self-reported boys or girls' TV viewing was predicted by self-reported parental TV viewing patterns. Child TV viewing (< 2 hours per day, 2-4 hours per day, and >4 hours per day) was the outcome with <2 hours per day as the reference group. The main exposure variable was parental TV viewing (< 2 hours per day, 2-4 hours per day, and >4 hours per day) with the model also adjusted for child BMI SDS, household IMD score, hours of daylight on the first day of data collection and parental BMI. As per the linear models, robust standard errors were used to account for the clustering of participants in schools. Analysis was performed in STATA version 10.0 (College Station, Texas) and alpha was set at 0.05.

## Results

Descriptive statistics for categorical variables are presented in Table 1. There were 431 parent-child dyads with some TV viewing questionnaire data and of these 352 (81.7%) of the parents were mothers. There were 340 dyads with complete accelerometer data. The sample was equally split between boys and girls. Around half of the children (53.4%) and parents (49.7%) reported watching less than 2 hours of television per day.

**Table 1:** Descriptive statistics for categorical outcomes

	N	% of overall sample
**Child and parent TV viewing data**		
Yes	431	41.5
No	605	58.5
**Child and parent accelerometer data**		
Yes	340	32.8
No	696	67.2

	**N**	**% of included sample**

Girls (valid TV viewing dyad)	215	49.9
Boys (valid TV viewing dyad)	216	49.7
Mothers (valid TV viewing dyad)	352	81.7
Father (valid TV viewing dyad)	79	18.3
**Child TV viewing per day**		
< 2 hours per day	230	53.4
2-4 hours per day	157	36.4
>4 hours per day	44	10.2
**Parent TV viewing per day**		
< 2 hours per day	214	49.7
2-4 hours per day	188	43.6
>4 hours per day	29	6.7

Descriptive statistics for continuous variables are presented in Table 2. The children engaged in an average of 35.3 minutes of MVPA per day with a mean of 36.9 minutes per day for parents. Independent sample t-tests indicated that the mean BMI SDS score of the children with valid parent and child accelerometer data was significantly lower (.36 vs. .55) than those excluded from the analysis for insufficient data (t = 2.34, p = .020) but there was no difference in IMD score for those included or excluded in the analyses. Conversely t-tests indicated that the IMD of parent-child dyads with valid self-reported TV viewing data was lower (.41 vs. .54) indicating that more affluent households were more likely to provide data (t = -3.06, p = .011) but there was no significant difference in child BMI SDS score.

**Table 2:** Descriptive statistics for continuous variables

	N	Mean	SD
Child BMI (kg/m^2^)	421	18.6	2.98
Child BMI SDS	419	.41	1.12
Child accelerometer counts per minute	340	548.2	172.0
Child sedentary minutes per day	340	552.3	103.1
Child MVPA minutes per day	340	35.3	16.7
Parent BMI (kg/m^2^)	381	25.25	5.33
Parent accelerometer counts per minute	340	481.3	549.2
Parent sedentary minutes per day	340	381.3	132.4
Parent MVPA minutes per day	340	36.9	27.3
IMD Score	418	.15	.12
Hours of daylight per day	419	11.42	2.33

Bivariate correlations between accelerometer derived child and parent physical activity behaviours are presented in Table 3. For girls, sedentary minutes was positively associated with parent sedentary minutes (r = .190, p = .012) and negatively associated with parent accelerometer counts per minute (r = -.150, p = .049). For boys sedentary time was positively associated with parent sedentary time (r = .178, p = .026).

**Table 3:** Correlations between parent and child accelerometer derived physical activity variables.

	Girls (n = 173)			Boys (n = 157)		
	**Child Sedentary minutes**	**Child MVPA minutes**	**Child CPM**	**Child Sedentary Minutes**	**Child MVPA minutes**	**Child CPM**

Parent Sed	.190*	.055	.041	.178*	.041	-.033
Parent MVPA	-.039	.084	.065	-.002	.044	.106
Parent CPM	-.150*	.081	.065	-.102	.002	.133

*p < 0.05

Linear regression models used to predict accelerometer-derived physical activity variables are presented in Table 4. For girls, parental sedentary minutes predicted child sedentary minutes (t = 2.43. p = .020) in a model that accounted for 12.1% of the variance. Parental CPM was not associated with girls' CPM and parental MVPA was not associated with girls MVPA. There were no statistically significant associations when the models were run for boys.

**Table 4:** Linear regression models predicting child accelerometer counts per minute, minutes of sedentary time per day and minutes of MVPA per day by gender

	Girls n = 139				Boys n = 129			
**Exposure Sedentary minutes**	**Coeff**	**95% CI**	**T**	**P**	**Coeff**	**95% CI**	**T**	**P**
	.204	.03 to .370	2.43	**0.020**	.097	-.04 to 0.23	1.45	0.155
								
	**Model R^2 ^= 0.121**				**Model R^2 ^= 0.093**			

**CPM**	.117	-.03 to 0.27	1.61	0.118	.146	-.07 to .37	1.35	0.185
								
	**Model R^2^= 0.116**				**Model R^2 ^= 0.109**			

**MVPA Minutes**	.034	-.10 to .17	0.53	0.598	.049	-.09 to .19	0.73	0.473
								
	**Model R^2 ^= 0.037**				**Model R^2 ^= 0.065**			

All models are adjusted for child BMI SDS score, Parental BMI, Home related index of multiple deprivation and hours of daylight on measurement day

The multinomial logistic regression models are shown in Table 5. For girls, the risk of watching more than 4 hours of TV per day, (when compared to the reference group of watching less than 2 hours of TV per day), was 3.67 times higher if the girl's parent watched 2-4 hours of TV per day (RR = 3.67, 95% CI = 1.08 to 12.42, p = 0.037). For boys, the risk of watching more than 4 hours of TV per day, (when compared to the reference group of watching less than 2 hours of TV per day), was 10.47 times higher if the boy's parent watched more than 4 hours of TV per day (RR = 10.47, 95% CI = 1.13 to 96.27, p = 0.038).

**Table 5:** Multi-nominal logistic regression models in which child's' TV viewing is predicted by parental TV viewing categories^1^

Girls n = 172	RR^2^	95% CI	Z	P
**Child 2-4 hours TV per day**				
Parent 2-4 hours TV per day	1.39	.85 to 2.26	1.32	0.186
Parent 4+ Hours of TV per day	1.49	.43 to 5.12	0.63	0.528
**Child 4+ hours TV per day**				
Parent 2-4 hours TV per day	3.67	1.08 to 12.42	2.09	**0.037**
Parent 4+ Hours of TV per day	3.05	.34 to 27.85	0.99	0.322
				**Pseudo R^2 ^= 0.041**

**Boys n = 174**	**RR**	**95% CI**	**Z**	**P**

**Child 2-4 hours TV per day**				
Parent 2-4 hours TV per day	1.09	.51 to 2.34	0.23	0.818
Parent 4+ Hours of TV per day	3.59	0.74 to 17.26	1.59	0.111
**Child 4+ hours TV per day**				
Parent 2-4 hours TV per day	0.72	0.26 to 2.07	-0.59	0.552
Parent 4+ Hours of TV per day	10.47	1.13 to 96.27	2.08	**0.038**
				**Pseudo R^2 ^= 0.070**

^1^Child TV < 2 hours per day is the referent category

^2^Relative Risk

Models are adjusted for child BMI SDS, Parental BMI, Home related index of multiple deprivation, and hours of daylight on measurement day

## Discussion

In this dataset, the overall sedentary time of parents and daughters was associated but there was no association for parents and sons. In the adjusted regression models there was no association between either the intensity (MVPA) or volume (CPM) of physical activity in which parents and children engage. Thus, girls who have parents who spend a lot of time being sedentary are more likely to be sedentary but there were no associations between parent and child physical activity for either boys or girls. The disparity between parent and child sedentary time for boys and girls is not immediately clear. It could be the case that girls are more likely to sit and engage in sedentary pursuits with their parents but we do not have data to explore this possibility. As such there is likely to be merit in further exploring why there is an association in sedentary time for girls and parents only and particularly if it is possible to work with parents to change girls' sedentary time. However, the absence of an association between parent and child physical activity suggests that strategies to promote parent and child physical activity together may not be fruitful at this age.

Higher parental TV viewing was associated with an increased risk that both boys and girls spent more than 4 hours per day watching TV. This finding is consistent with our previous research in which we have shown that children who live in a TV watching promoting household (TV is on when the child comes home from school and meals are eaten in front of the TV) are more likely to exceed the AAP recommendation [44]. Collectively, these findings suggest that parent and child TV viewing are related and that developing strategies to change the home TV environment may be important for reducing children's TV viewing.

This paper has significantly advanced current knowledge by providing a unique combination of both objective and self-reported data to document the associations between parent and child physical activity, sedentary time and TV viewing behaviours using robust measures and after adjustment for SES and demographic factors. However, the study has a number of limitations that need to be recognised. Firstly, parent and child dyad TV viewing data were only available for 431 children and the accelerometer data only met the inclusion criteria for 340 dyads. As noted above, children with valid accelerometer data had lower BMI values than those excluded while the children included in the TV viewing analyses tended to live in less deprived households than those who did not provide data. Caution is therefore required when interpreting the results, as it may be that associations are not comparable in less affluent households where, in the UK, there are higher levels of childhood and adult obesity and TV viewing [48-50]. Moreover, we have not been able to control for other potential confounders such as the geographical location of the home. Therefore, re-examining these issues in a dataset with a greater representation of participants from lower SES groups and a wider array of neighbourhood level variables is warranted.

It is important to recognise that parents were selected on the basis that they self-identified as the primary carer for their child and this yielded a sample that was over 80% female. Davison and colleagues [24] have previously reported that parental facilitation of 9 year old girls' physical activity differed by parental gender and therefore it may also be the case that direct modelling also differs by parental and child gender. In light of the small number of fathers who provided data it is not possible to examine if associations differed by parental gender or if associations were stronger in same sex dyads. The relative absence of paternal data may reflect contemporary care giver roles whereby mothers are generally more likely to complete surveys about their child than fathers. As such there is a need for more information from fathers in future datasets.

Although parent and child sedentary time were associated, the model accounted for only 12.1% of the variance. Similarly, the pseudo R^2 ^for the multi-nominal regression models accounted for less than 8% of the variance in children's TV viewing. Moreover, although parental TV viewing was associated with an increase in the relative risk that children spent more than 4 hours per day watching TV, the 95% confidence intervals for statistically significant findings (RR = 3.67, 95% CI = 1.08 to 12.42, and RR = 10.47, 95% CI = 1.13 to 96.27) were very large indicating a lack of precision in associations. These findings therefore reinforce the need to further examine these associations in other datasets and search for potential predictors of youth behaviour such as parental facilitation of activity and TV viewing.

It has frequently been suggested that parental modelling of activity behaviour is likely to be central in promoting physical activity among children [23-25]. Understanding the importance of parental modelling of physical activity has previously been difficult because studies have relied on less precise assessments of physical activity such as physical activity recall surveys [51]. The absence of an association between parent and child physical activity could be a function of the child's age, with 10-11 years of age being a period when children's cognitive decision making abilities increase and they begin to assert a degree of independence from their parents [25,52,53]. As such, it may be the case that there are associations among younger children. The absence of a direct association in physical activity is coherent with our qualitative work in which parents reported that they rarely engaged in physical activity with their child, spending more time arranging transport and supervision for their child's physical activity [25,26]. Collectively, these findings suggest that parental influence on the physical activity of British 10-11 year olds is likely to be facilitative in nature and not by modelling or copying of behaviours. Attention should therefore focus on understanding this association and identifying effective strategies for parents to use to enable their children to be as active as possible.

The findings presented here raise a number of issues for both public health practice and research. Many adults, and particularly mothers, feel unable to engage in physical activity because they feel obligated to prioritise their time toward family caretaking responsibilities and may also have low levels of perceived physical activity competence and thus feel limited in how they can be physically active role models for their children [54-56]. Our data indicate that parents don't need to be active to influence their child's physical activity. Thus, we would suggest that a key public health message should be that all parents, regardless of their own activity level should be encouraged to promote physical activity and reduce sedentary time for their child.

The data presented here indicate that on the average, parents are engaging in similar amounts of MVPA as children but it seems that this is not activity in which parents are active with their children. In terms of future research this suggests that promoting strategies that focus on helping parents and children to be active on their own should be developed alongside those that promote parents and children being physically active together. Secondly, as our data indicate that parent-child co-participation in activity is unlikely to be a source of parental influence, any existing parental influence is likely to be a function of parental facilitation of physical activity. Therefore, there is a need to examine the nature of facilitative influence and particularly if parents can help their children to be active by encouraging active travel to school or promoting outdoor free-play in safe areas close to home [25].

## Conclusions

The data presented in this paper demonstrate that there was an association between the sedentary time of girls and their mothers. Additionally, higher parental TV viewing was associated with higher child TV viewing among both boys and girls. There were no associations between the time that parents and children spend engaged in physical activity.

## Competing interests

The authors declare that they have no competing interests.

## Authors' contributions

The study was conceived by RJ, KRF, ASP and JLT. RJ performed all analysis and wrote the first draft of the paper. All authors provided key intellectual input into the paper and edited the paper.

## Pre-publication history

The pre-publication history for this paper can be accessed here:

http://www.biomedcentral.com/1471-2458/10/194/prepub
